# Retraction Note: Analytical investigations of propagation of ultra-broad nonparaxial pulses in a birefringent optical waveguide by three computational ideas

**DOI:** 10.1038/s41598-025-86472-3

**Published:** 2025-01-21

**Authors:** Yuanyuan Liu, Jalil Manafian, Gurpreet Singh, Naief Alabed Alkader, Kottakkaran Sooppy Nisar

**Affiliations:** 1 Department of Mathematics and Artificial Intelligence, Lyuliang University, Lüliang, China; 2 Department of Applied Mathematics, Faculty of Mathematical Sciences, University of Tabriz, Tabriz, Iran; 3 Natural Sciences Faculty, Lankaran State University, 50, H. Aslanov Str., Lankaran, Azerbaijan; 4 Department of Applied Sciences, Chitkara University Institute of Engineering and Technology, Chitkara University, Punjab Rajpura, India; 5 Department of Sustainable Finance, Plekhanov Russian University of Economics, 117997 Moscow, Russia; 6 Department of Mathematics, College of Science and Humanities in Alkharj, Prince Sattam bin AbdulazizUniversity, Alkharj, 11942 Saudi Arabia

Retraction of: *Scientific Reports* 10.1038/s41598-024-56719-6, published online 15 March 2024

The Editors have retracted this Article. After publication, concerns were raised about the validity of the research presented in this article. The method proposed for obtaining solutions to the coupled non-linear partial differential equations (PDEs) presented in Eq. (1), described in the Sections The method and General properties of generalized G‑expansion method, is not clearly defined and is likely incorrect. Important steps in the derivation of solutions to these PDEs have either been omitted or presented together with a derivation of solutions to a different, and more general, problem, and therefore not immediately relevant. Furthermore, several variables in this derivation, e.g. α, m1, m2, are not correctly defined and have no clear mathematical meaning.

The Authors shared the source code for this research with the Journal. However, an expert peer review found considerable differences between the method described in the paper and that implemented in the source code.

All Authors disagree with this retraction.

